# Case Report: Second Report of Joubert Syndrome Caused by Biallelic Variants in IFT74

**DOI:** 10.3389/fgene.2021.738157

**Published:** 2021-09-01

**Authors:** Ke Zhongling, Li Guoming, Chen Yanhui, Chen Xiaoru

**Affiliations:** ^1^Fujian Medical University Union Hospital, Fuzhou, China; ^2^Guankou Hospital of Xiamen Jimei District, Xiamen, China

**Keywords:** Joubert syndrome, ciliopathy, IFT74, developmental delay, polydactyly, cleft lip

## Abstract

Joubert syndrome (JBTS) is a rare ciliopathy characterized by developmental delay, hypotonia, and distinctive cerebellar and brain stem malformation called the molar tooth sign (MTS). We reported a 15-month-old female with dysmorphic features (flat nasal bridge, almond-shaped eye, and a minor midline notch in the upper lips), hypotonia, polydactyly, development delay, and MTS. Whole exome sequencing revealed biallelic heterozygous mutations c.535C>G(p.Q179E/c.853G>T) (p.E285^*^) in *IFT74*, which were inherited from the parents. So far, only one article reported JBTS associated with *IFT74* gene mutation, and this is the second report of the fifth patient with JBTS due to variants in *IFT74*. All five patients had developmental delay, postaxial polydactyly, subtle cleft of the upper lip, hypotonia, and MTS, but notably without renal and retinal anomalies or significant obesity, and they shared the same mutation c.535C>G(p.Q179E) in *IFT74*, and c.853G>T(p.E285^*^) that we found was a new mutation in *IFT74* that related with Joubert syndrome. Those findings highlight the need for the inclusion of *IFT74* in gene panels for JBST testing.

## Introduction

Joubert syndrome (JBTS, OMIM: P213300) is a rare, autosomal recessive ciliopathy characterized by three primary findings: a distinctive cerebellar and brain stem malformation called the molar tooth sign (MTS), hypotonia, and developmental delay (Parisi et al., 2017). About 35 ciliopathy-related genes are known to cause JBTS (Radha Rama Devi et al., 2020); those genes encode proteins localized to the pericilia, whose dysfunction would alter cilia composition or signaling (Parisi, 2019). The Intraflagellar transport(IFT) complex is the main module for regulating cilia composition, which consists of IFT-A and IFT-B, and *IFT74* is required for the stabilization of IFT-B (Brown Jason et al., 2015). However, the relationship between JBST and IFT is rarely studied; thus far, only three studies have researched the correlation between JBST and IFT (Halbritter et al., 2013; Bachmann-Gagescu et al., 2015; Luo et al., 2021). In February this year, Luo et al. (2021) reported for the first time that JBST could be caused by *IFT74* mutation. This paper was the second report of the fifth case of *IFT74*-associated JBTS; we found a new mutation in *IFT74*-associated JBTS and present new craniofacial dysmorphisms, which helped to expand the clinical phenotype and genotype of this syndrome.

## Case Report

The proband was a 15-month-old female who was referred to our department because of developmental delay. She was the first child of non-consanguineous Chinese parents.

**Figure 1:**
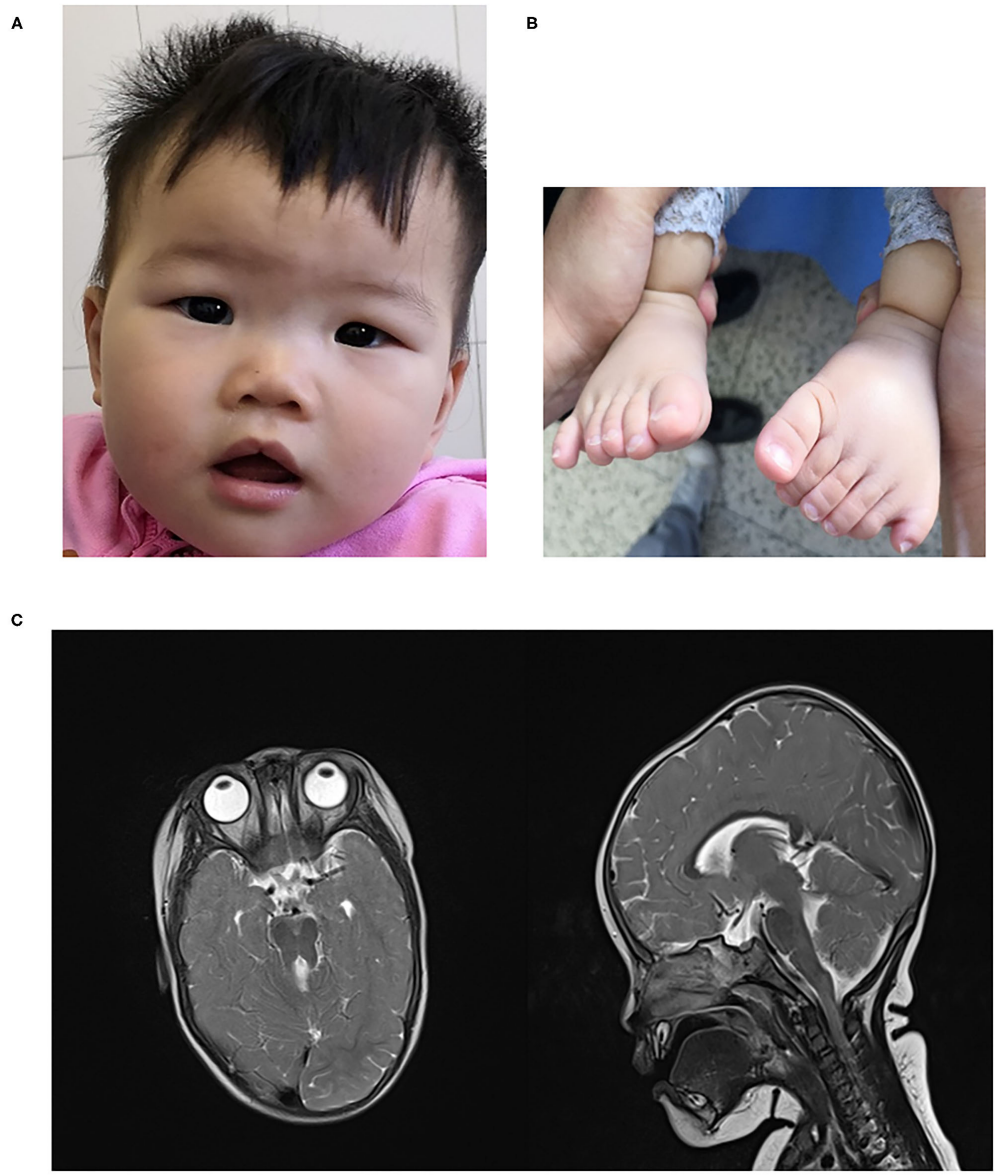
**(A)** craniofacial dysmorphism **(B)**. polydactyly **(C)** MTS in brain MRI.

She was delivered at term via spontaneous vaginal delivery to a 35-year-old mother after an uncomplicated pregnancy. Birth weight was 3,400 g. Immediately after birth, craniofacial dysmorphisms with a flat nasal bridge, almond-shaped eye, and a minor midline notch in the upper lips and postaxial polydactyly of the hands and feet were noticed (Figures 1A,B). She achieved rising head and sitting at 3 and 8 months old, respectively. Delayed motor development was noticed when she couldn't crawl at 1 year, and slight hypotonia of lower limbs was found. She began speaking at 1 year with slowly progressive. At the age of 9 months, she underwent a hand polydactyly excision.

There were no other family members with the presence of birth defects, developmental delay, and/or any other neurological disorders.

On examination at 15 months of age, she can stand with the assistant and speak several words. Her weight was 13 kg, height was 82 cm, body mass index (BMI) was 19.3 kg/m^2^. Head circumference was 46 cm. Investigations showed that blood count, urine routine test, biochemical test, thyroid hormones, 25 hydroxyvitamin D3 -were normal. No significant abnormalities were seen in cardiac and abdominal ultrasound examinations. An audiology evaluation was normal. Fundoscopic examination showed no retinal dystrophy, and cranial MRI showed MTS (Figure 1C). Neuropsychological development assessment was performed at the age of 16 months; the development quotient (DQ) assessment was as follows: total (64)- gross motor (52)—fine motor (64)—adaptability (64)—language ability (55)—social ability (67).

**Figure 2:**
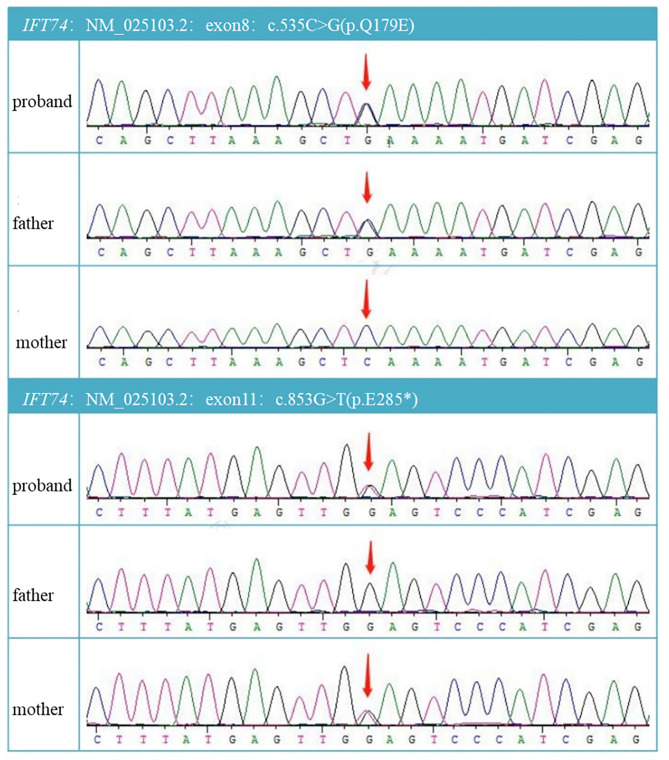
DNA electrophoregram with the c.535C >G in exon8 and c.853G>T in exon11.

**Figure 3:**
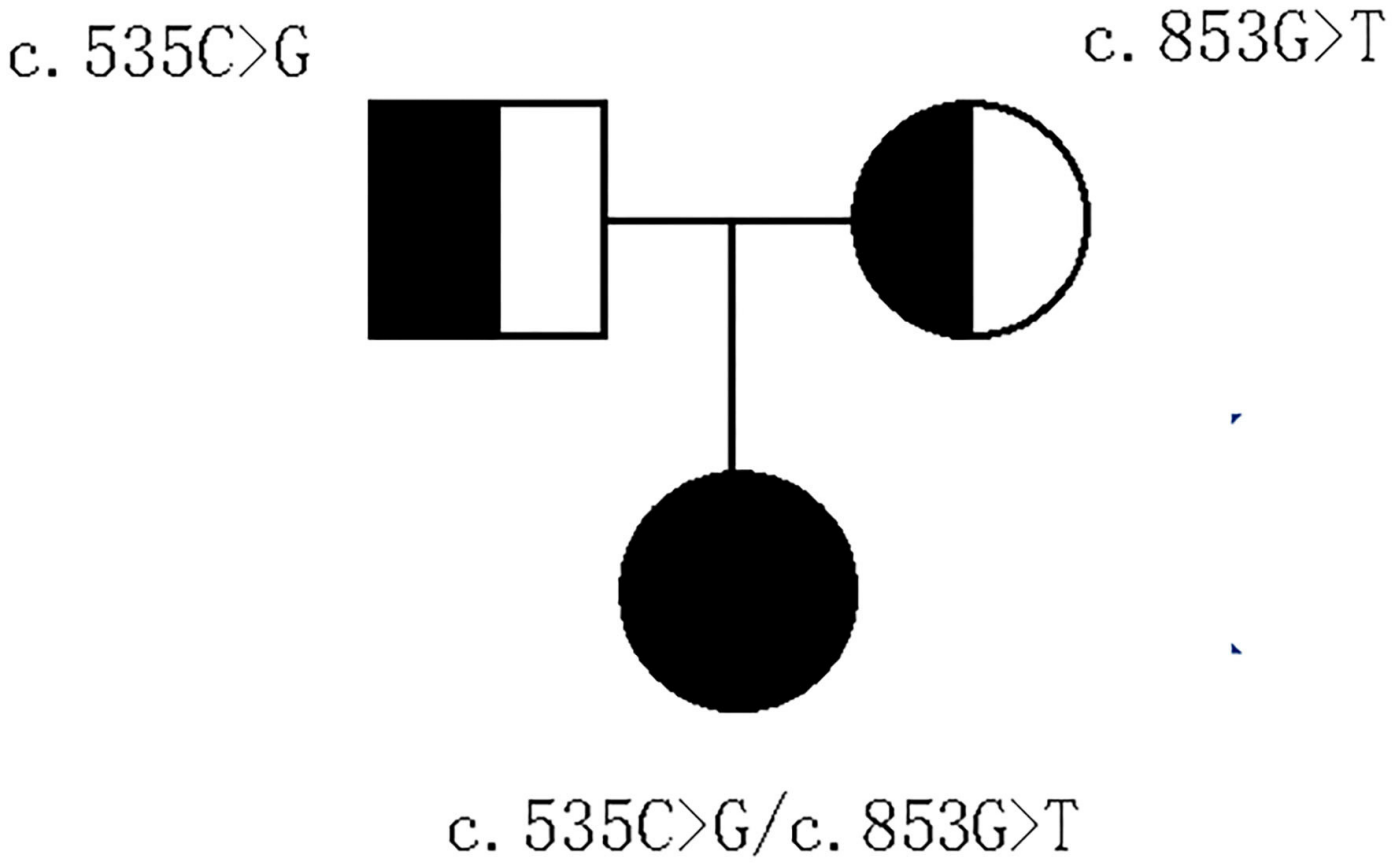
Pedigree of the family.

With parental consent, blood was collected from the child and parents, and whole-exome sequencing was performed. Genomic DNA was extracted from the blood sample with the Blood Genomic DNA Mini Kit following manufacturer's guidelines (CWBIO). The whole-exome library was prepared using the SureSelect Human All Exon V6 (Agilent) and KAPA Hyper Prep Kit (KAPA) following the manufacturer's protocol. All sequencing was performed on the Nova seq 6,000 platform (Illumina) (Testing Service Company: The medical laboratory of Nantong Zhongke, China). Alignment and variant calling were performed with an in-house bioinformatics pipeline. Variants with a minor allele frequency of <0.05 in population databases and expected to affect coding/splicing of the protein or were present in the Human Gene Mutation Database (HGMD) (Stenson et al., 2003) were included in the analysis. The American College of Medical Genetics and Genomics (ACMG) Standards and Guidelines for the interpretation of sequence variants were followed in this study.Two heterozygous variants in *IFT74*, NM_025103.4,c.535C>G(p.Q179E)/c.853G>T(p.E285^*^) were identified. Sanger sequencing showed the variants were inherited from the parents, confirming that the variants were indeed biallelic (Figures 2, 3). c.535C>G(p.Q179E) was predicted to be “disease causing” with a score of 0.99 (MutationTaster), “benign” with a score of 0.126 (PolyPhen-2), and “tolerated” with a score of 0.14 (SIFT), and c.853G>T(p.E285^*^) was predicted to be “disease causing” with a score of 1 (MutationTaster).

Accroding to ACMG, c.535C>G(p.Q179E) was pathogenic(PS1+PS3),while c.853G>T(p.E285^*^) was uncertain significant(PM2).

## Literature Review

**Figure 4:**
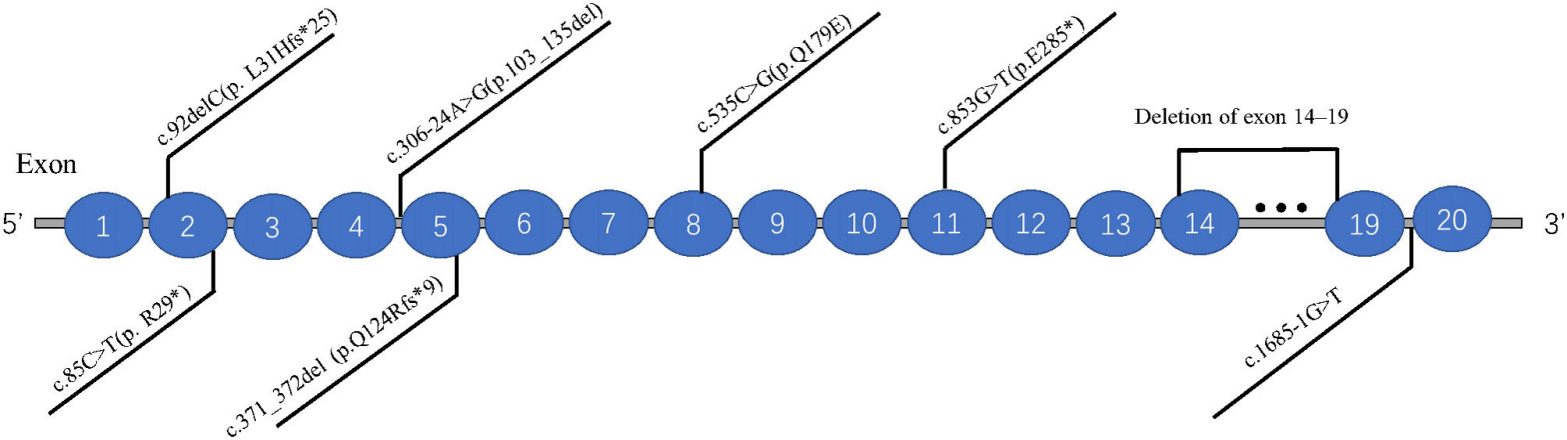
Distribution of eight *IFT74* gene mutations (transcript: NM_025103.4).

The databases of Pubmed, EMCC, EBSCO, and Cochrane Library were searched with the keywords of “Joubert syndrome” and “*IFT74*,” and only one related study was found (Luo et al., 2021). And there were three articles (Lindstrand et al., 2016; Kleinendorst et al., 2020; Mardy et al., 2021) about *IFT74* gene mutation related to Bardet-Biedl syndrome (BBS). The clinical features and mutations of all these cases were summary in Table 1, Figure 4.

**Table 1:** Clinical features of patients with biallelic *IFT74* variants.

**Featrue**	***IFT74*-JBST case 5 (our case)**	***IFT74*-JBST Case 1 (Luo)**	***IFT74*-JBST Case 2 (Luo)**	***IFT74*-JBST Case 3 (Luo)**	***IFT74*-JBST Case 4 (Luo)**	***IFT74*-BBS case 1 (Lindstrand)**	***IFT74*-BBS case 2 (Kleinendorst)**	***IFT74*-BBS case 3 (Mardy)**
Age	1 year 3 months	13 years 5 months	1 year 11 months	4 years 6 months	7 years 2 months	36 years	11 years	6 years
Gender	Female	Female	Female	Male	Male	Male	Female	male
Ethnicity	Chinese	Chinese	Chinese	Chinese	Chinese	NA	Dutch	mixed race
Variant 1	c.853G>T (p.E 285^*^)	c.92delT (p.L31Hfs^*^25)	c.92delT (p. L31Hfs^*^25)	c.306-24A>G (p.103_135del)	c.85C>T (p. R29^*^)	Deletion of exon 14–19	c.371_372del (p. Q124Rfs^*^9)	c.1685-1G > T
Variant 2	c.535C>G (p. Q179E)	c.535C>G (p. Q179E)	c.535C>G (p. Q179E)	c.535C>G (p. Q179E)	c.535C>G (p. Q179E)	c.1685-G>T	c.1685-1G>T	c.1685-1G > T
Height (cm)	13	148.5	78	104	116	NA	162.7	126.7
Weight (kg)	82	40	8.9	14	17.8	NA	70.94	36.9
BMI (kg/m2)	19.33	18.14	14.63	12.94	13.23	NA	26.80	22.99
MTS	+	+	+	+	+	–	–	–
Oculomotor apraxia	–	+	+	+	+	NA	–	–
Respiratory abnormality	–	–	+	+	–	NA	–	–
Hypotonia	+^a^	+	+	+	+	NA	–	–
Retinal involvement	–	–	–	–	–	Retinitis pigmentosa	Rod–cone dystrophy	Early retinal dystrophy
Optic nerve hypoplasia/RNFL defect		+/+	+/+	–/–	–/+	NA/NA	–/–	+
Renal involvement	–	–	–	–	–	–	–	–
Liver involvement	–	–	–	–	–	NA	–	–
Postaxial polydactyly	+	+	+	+	+	+	+	+
Developmental delay	+	+	+	+	+	–	–^b^	+
Intellectual disability	Mild	–	Moderate	Mild	Mild	+	–	–
Hypogonadism (in males) or genital abnormalities (in females)	–	–	–	–	–	Hypogonadism	–	–
Craniofacial dysmorphisms	Midline notch in the upper lip Fat, nasal bridge, almond-shaped eye	Midline cleft lip	Midline notch in the upper lip	Midline cleft lip	Midline notch in the upper lip	Microcephaly	Macrocephaly	Normal
Truncal obesity	–	–	–	–	–	+	+	+
Diabetes mellitus	–	–	–	–	–	–	–	–
Behavioral problem	–	–	Self-mutilation	–	–	–	–	–

*JBTS, Joubert syndrome; BBS, Bardet–Biedl syndrome; BMI, body mass index; MTS, molar tooth sign; NA, not available or not mentioned; RNFL, retinal nerve fiber layer; –, not present; +, present*.

a *slight hypotonia of lower limbs*.

b *Only speech delay in childhood*.

## Discussion

This is the second report of *IFT74* variants causing a JBTS, validating *IFT74* as a JBTS gene. So far, only one previous report of four cases with JBTS caused by biallelic *IFT74* variants has been published by Luo et al. (2021) this year. Luo et al. described four affected individuals from three non-consanguineous families presenting with MTS on brain MRI, delay in global developmental milestones, postaxial polydactyly, and subtle cleft of the upper lip, all the affected individuals shared one missense variant (p.Q179E), and the pathogenic effects of the variant were evaluated by animal model, the *IFT74* was identified as a JBTS-associated gene. Our patient's phenotype was consistent with Luo et al.'s report and also shared the missense variant (p.Q179E); however, the other mutation, c.853G>T(p.E285^*^), found in our patient had not been reported in Joubert syndrome. The patient also had craniofacial dysmorphisms with a flat nasal bridge and almond-shaped eye, and these features have not been previously described in patients with a mutation of *IFT74*.

*IFT74* gene is located on chr9: 26956412-27066134, with 20 exons (ensembl, 2021). *IFT74* encodes a core intraflagellar transporter protein (IFT) that belongs to a multiprotein complex involved in the transport of ciliary proteins along axonemal microtubules. This protein binds to intraflagellar transporter protein 81 and transports microtubule proteins within the cilium, which is required for ciliogenesis (Ncbi.nlm.nih.gov., 2021). Although JBTS is known to be a recessive and heterogeneous ciliopathy (Bachmann-Gagescu et al., 2020), very little attention was paid to the relationship between *IFT74* and JBTS. Until this year, Luo et al. (2021) comprehensively analyzed the cohort of 4 patients, all of which carried pathogenic mutations in the *IFT74* and showed similar phenotypes, and identified *IFT74* as a JBTS-associated gene.

Previous studies have suggested that *IFT74* gene mutation is mainly associated with BBS, which is characterized by obesity and related complications, retinal cone-rod dystrophy, postaxial polydactyly, cognitive impairment, and renal malformations and/or renal parenchymal disease (Forsythe et al., 2018). Combined with previous reports and our findings, we found that the patients with biallelic variants in *IFT74* all suffered from postaxial polydactyly and none of them had renal involvement, and the cleft lip was a special craniofacial dysmorphism in *IFT74* related JBTS while retinal dystrophy and truncal obesity could be seen in all *IFT74* related BBS. Intellectual disability was common in *IFT74* related JBTS but with a mild or moderate degree. The mutations of *IFT74* with JBTS include 2 non-sense mutations,2 frameshift mutations, 1 splice mutations, 1 missense mutation, and were distributed in exons 2, 5, 8, 11, and intron 4. *IFT74* mutations with BBS include 1 complete gene deletions and 1 splice mutations, distributed in exons 14–19 and intron 19. Those showed that the *IFT74* mutation before exon 11 were associated with JBST, while mutations after it was associated with BBS. Although the mutation was in the same gene, mutations in different locus regions may result in various protein changes, leading to these two distinct syndromes. The most common mutation type is c.535C>G (p. Q179E), which was present in all the *IFT74* related JBST; mechanistic studies suggested that pathogenic variants in *IFT74* lead to defects in cilia length, ciliogenesis, cilia composition, and Hh signaling (Luo et al., 2021), whether c.535C>G (p. Q179E) is hot spot mutations or not need to be confirmed by future data. The mutation c.853G>T(p.E285^*^) found in this patient had not been reported in Joubert syndrome, and there was no research on the pathogenesis of this mutation, but according to our clinical finding, it play a vital part in the JBTS, suggesting the loss of function due to this mutation may be pathogenic, further studies on the alter function of this mutation are needed.

In conclusion, our study reported a compound heterozygous mutation in the *IFT74* gene [c.535C>G(p.Q179E)/c.853G>T(p.E285^*^)] in a Chinese family with JBTS.As far as we know, this is the second report of the fifth case in *IFT74* mutation-related JBST. This patient helps to expand and clarify the clinical spectrum of *IFT74*-related JBST, and the cases with *IFT74* mutation are summarized in Table 1. The *IFT74* mutation-related JBST manifested developmental delay, postaxial polydactyly, subtle cleft of the upper lip, hypotonia, and MTS on brain MRI, but notably without renal and retinal anomalies or significant obesity. Our case also presents new craniofacial dysmorphisms of the flat nasal bridge and almond-shaped eye and the new mutation c.853G>T(p.E285^*^) in JBST.All our findings highlight the need for the inclusion of *IFT74* in gene panels for JBST testing.

## Data Availability Statement

The raw data supporting the conclusions of this article will be made available by the authors, without undue reservation.

## Ethics Statement

The studies involving human participants were reviewed and approved by Fujian medical university union hospital. Written informed consent to participate in this study was provided by the participants' legal guardian/next of kin. Written informed consent was obtained from the individual(s), and minor(s)' legal guardian/next of kin, for the publication of any potentially identifiable images or data included in this article.

## Author Contributions

KZ contributed to the acquisition, analysis of data, and writing the first draft. CY contributed to the conception of the work and revised the paper. LG contributed to collect the data and communicated with the pateints family. CX contributed to search information of this disease.

## Conflict of Interest

The authors declare that the research was conducted in the absence of any commercial or financial relationships that could be construed as a potential conflict of interest.

## Publisher's Note

All claims expressed in this article are solely those of the authors and do not necessarily represent those of their affiliated organizations, or those of the publisher, the editors and the reviewers. Any product that may be evaluated in this article, or claim that may be made by its manufacturer, is not guaranteed or endorsed by the publisher.
